# Closing Time! Examining the Impact of Gender and Executive Branch Policy Makers on the Timing of Stay-at-Home Orders

**DOI:** 10.1017/S1743923X20000264

**Published:** 2020-05-29

**Authors:** Laine P. Shay

**Affiliations:** Texas A&M University–Corpus Christi

**Keywords:** State politics, health policy, executive branch politics, bureaucratic politics, gender politics

## Abstract

The 2019–20 coronavirus pandemic has significantly altered lives across the globe. In the United States, several states attempted to manage the pandemic by issuing stay-at-home orders. In this research note, I examine whether the gender of state policy makers in the executive branch might impact a state's adoption of a stay-at-home order. Using event history analysis, I find that the governor's gender has no impact on the likelihood of a state adopting a stay-at-home order. However, I find that gender plays a significant role for agency heads. Specifically, my analysis shows that states with a female-headed health agency tend to adopt stay-at-home orders earlier than states with a male administrator. These findings shed light on how female leadership in the executive branch may impact public policy regarding COVID-19.

The 2019–20 coronavirus pandemic has significantly altered lives across the globe. In the United States, several states attempted to manage the crisis by implementing some form of stay-at-home order. While varying by state, stay-at-home orders allow citizens to leave their homes only for essential business. Schools, gyms, restaurants, and other businesses were forced to close their doors. States implemented these policies in drastically different manners. For instance, on March 19, California was the first state to implement a stay-at-home order. Dr. Sonia Angell, director of the Department of Public Health, warned that the policy was necessary to “bend the curve, and disrupt the spread of the virus.”^1^ Other states, such as Iowa, had yet to implement a stay-at-home order as of May 1, 2020. This variation raises the question: what factors account for when a state adopts a stay-at-home order?

Some journalists suggest that female leaders respond differently than men in managing COVID-19 (e.g., Anderson 2020). Recent research in gender politics suggests that a policy maker's gender may indeed impact their decisions to handle COVID-19 (Atkinson and Windett 2019; Dolan 2002; Swers 2002). In this research note, I examine whether the gender of key executive branch officials helps explain when a state adopts a stay-at-home order. Using event history analysis, I find that the gender of the governor has no impact on when a state adopts a stay-at-home order, but states with a female-headed health agency tend to adopt a stay-at-home order more quickly. These findings shed light on the importance of female leadership within the executive branch in managing COVID-19.

## LINKING THE GENDER OF STATE EXECUTIVE BRANCH POLICY MAKERS TO STAY-AT-HOME ORDERS

A growing literature explores the impact gender has on state policy makers. Several studies show that female policy makers tend to be more liberal than their male counterparts (Burrell 1994; Swers 2002; Thomsen 2018). This pattern emerges because conservatives tend to embrace more traditional gender roles and focus less on gender equality. Further, women are more likely to be recipients of social policy (Orloff 1993) and hence more likely to embrace a liberal economic agenda. If female policy makers favor a more activist state, then they may advocate for stay-at-home orders more strongly. Implementing such orders requires government action, a decision that might not be ideologically appealing to conservative policy makers.

Research also demonstrates differences in policy priorities between male and female policy makers. Studies show that certain policy areas are characterized as “women's issues.” Such policies include the environment, social welfare, and, relevant to my research, health care (Atkinson and Windett 2019; Barnes 2016; Clayton and Zetterberg 2018). This research suggests that women feel empowered to address issues in these policy areas because they are more female dominated (Karpowitz and Mendelberg 2014). Similarly, Clayton and Zetterberg (2018) find that an increase in female parliamentary representation is associated with an increase in health spending. If men place less priority on health policy, then we should expect gender differences among policy makers in advocating for stay-at-home orders.

Research suggests that gender may matter in advocating for stay-at-home orders, but several policy makers have influence over the timing of such orders. The governor obviously has significant influence over the timing of stay-at-home orders, ultimately deciding, in most states, if and when such an order is adopted. Thus, we might observe earlier stay-at-home orders in states with female governors.

Bureaucratic actors could also influence the timing of stay-at-home orders. Several studies show that gender can impact the bureaucracy (Dolan 2002; Park 2012; Wilkins 2007). For instance, Dolan (2002) finds that female administrators are more likely to support increased health care spending. I suggest that the gender of the administrator who heads the state's public health agency could play a key role in the timing and adoption of stay-at-home orders. These administrators are responsible for advising the governor on issues pertaining to health care in the state and interpret policy information for the state's chief executive. Of course, governors have other advisers to rely on for managing the pandemic.

Further, the influence of a state's health agency head on the governor likely varies by state. However, these officials have access and expertise that they can use to influence the governor on decisions related to the pandemic. Several journalists have noted that Ohio's director of health, Dr. Amy Acton, has significant sway over Governor Mike DeWine and his COVID-19 policy decisions. In fact, Dr. Acton played a pivotal role in crafting Ohio's stay-at-home order (Staver 2020). Thus, I expect that states with female-headed health agencies might be more likely to quickly adopt a stay-at-home order than states with male-headed health agencies.

## RESEARCH DESIGN

Since my research question concerns when a state adopts a stay-at-home order, event history analysis is the appropriate approach. I utilize a discrete-time model (Box-Steffensmeier and Jones 1997). Consistent with this approach, my unit of analysis is the state and day. My analysis starts on March 14, the day after President Donald Trump declared a national emergency due to COVID-19. It ends on April 18, a few days after COVID-19 cases peaked in the United States. The last state to adopt a stay-at-home order in my data set is South Carolina on April 7.

### Dependent Variable

The dependent variable for this analysis captures whether a governor has adopted a stay-at-home order. Specifically, it is a binary indicator of whether a state adopted an order on a given day (Lee 2020).

Consistent with event history analysis, once a state adopts a stay-at-home order, it is dropped from my data set. I estimate my coefficients with a logistic regression estimator and with a random effect on each state.

### Key Independent Variables

I have two primary independent variables: female governor and female health administrator. The female governor variable is coded as whether the incumbent governor in a state is a female (1) or male (0). The female health administrator variable is coded as whether the administrator of the state's public health agency is a female (1) or male (0).

### Additional Explanatory Variables

As suggested by Box-Steffensmeier and Jones (1997), I include a duration dependence variable, which is a count of the total number of days since President Trump declared a national emergency. I square this variable. I also control for other variables that might influence when a state adopts a stay-at-home order. Additional details describing these control variables can be found in the online appendix.^2^

## FINDINGS

The results from my logistic regression model can be found in Table 1. A positive and significant coefficient indicates that an increase in the corresponding variable is associated with a state more quickly adopting a stay-at-home order. The female health administrator variable is significant and positively signed. This indicates that states with a female administrator heading the state's public health agency tend to more quickly adopt a stay-at-home order than public health agencies that are headed by a male administrator.

**Table 1.** Event history analysis of COVID-19 stay-at-home orders

	Coefficient	% Change in Odds
Variable	(SE)	
**Female health administrator**	1.246*	**+248%**
	**(0.508)**	
**Female governor**	**–0.613**	**~**
	**(0.680)**	
Democratic governor	1.635*	+413%
	(0.523)	
Democratic electorate	0.065*	+7%
	(0.033)	
Upcoming governor election	1.572*	+382%
	(0.605)	
Total interest groups	–1.287*	–72%
	(0.594)	
Health industry interest group	0.045	~
	(0.119)	
Hotel/small business industry interest groups	–0.431*	–35%
	(0.206)	
New fatalities	0.094*	+10%
	(0.044)	
New cases	0.000	~
	(0.001)	
Income per capita	–0.065*	–6%
	(0.029)	
Urban	–0.055*	–5%
	(0.028)	
Population density	0.003*	3%
	(0.001)	
Percentage African American	0.010	~
	(0.033)	
Percentage Hispanic	0.065*	+7%
	(0.031)	
South	–0.843	~
	(0.757)	
Duration dependence	0.941*	+156%
	(0.195)	
Duration dependence squared	–0.022*	–2%
	(0.006)	
Constant	–1.379	~
	(5.852)	
BIC	364	
Observations	899	

*Notes:* Coefficients are estimated with a logistic regression estimator. A random effect is included on each state. Bold entries indicate key independent variables. *p ≤.05 (all one-tailed tests).

To ease the substantive interpretation, the coefficients can be translated into the percentage change in the odds. The odds of adopting an order are 248% higher for a state with a female-headed health agency than the odds for a state with a male administrator. It appears that the gender of the governor has little effect on the likelihood of a state adopting a stay-at-home order, at least once other factors are taken into consideration.

## CONCLUSIONS AND IMPLICATIONS

In this research note, I explore whether the gender of state executive branch policy makers has any influence on when a state adopts a stay-at-home order pertaining to COVID-19. Using event history analysis, I find that the gender of the governor has no impact on the timing of stay-at-home orders. However, I do find that public health agencies that are headed by a female administrator correspond with states more quickly adopting a stay-at-home order. These results imply that the gender of bureaucratic administrators advising and informing the governor can play a key role in important policy decisions and debates.

Before concluding, a few limitations of this study need to be highlighted. First, it is important to note that the stringency and restrictions of a stay-at-home order vary greatly by state. Future research should examine this issue in greater detail. Second, a few cities and counties implemented their own stay-at-home orders before the governor. Additional research should examine the factors that cause municipal governments to implement such policies.

These findings speak to the importance of female representation in the executive branch and bureaucracy, suggesting that the gender of bureaucratic officials can indeed impact policy. This comes at a key time when some politicians have argued that “diversity isn't important” in terms of bureaucratic officials (Ganim 2018). My findings show that gender diversity is indeed important, and it can impact policy. Public health experts argue that stay-at-home orders save lives and minimize the negative consequences of COVID-19 (Godoy 2020). My results suggest that women's leadership roles within the executive can lead to earlier stay-at-home orders and provide insight into the relationship between female executive branch leadership and health policy.

## Footnotes

1. Order of the State Public Health Officer, March 19, 2020, https://www.cdph.ca.gov/Programs/CID/DCDC/CDPH%20Document%20Library/COVID-19/Health%20Order%203.19.2020.pdf (accessed May 28, 2020).

2. The replication data for this article are available on Laine Shay's website: https://sites.google.com/site/lainepshay/home/research.

## SUPPLEMENTARY MATERIAL

For supplementary material accompanying this paper visit http://dx.doi.org/10.1017/S1743923X20000264. click here to view supplementary material
