# Supplementary material for: Closing Time! Examining the Impact of Gender and Executive Branch Policy Makers on the Timing of Stay-at-Home Orders
Source: Politics & Gender. 2020 May 29:1–8. doi: 10.1017/S1743923X20000264 (PMC7360953; doi:10.1017/S1743923X20000264)
Supplement: Supplementary file 1 [file S1743923X20000264sup001.docx]

Supplemental Appendix for
“Closing Time! Examining the Impact of Gender and Executive Branch Policymakers on the Timing of Stay-at-Home Orders”

Laine P. Shay

Texas A&M-Corpus Christi

Laine.Shay@utah.edu

This appendix includes additional details about the data and robustness tests. The appendix proceeds with two sections. The first section describes the explanatory variables in greater detail. The second section shows the results with alternative transformations of the duration dependence variable.

**Appendix A: Description of Control Variables**

In this section, I describe how I measure each of the control variables that are included in my model. Listed below are my control variables.

**Female Governor**: Indicating whether the governor is a female (1) or male (0).

**Female Health Administrator**: Indicating whether the state’s public health agency head is a female (1) or male (0). This data primarily comes from the following website: https://www.astho.org/Directory/. I also searched for these administrators through state websites.

**Democratic Governor**: Indicates whether the governor is a Democrat (1) or a Republican (0).

**Democratic Electorate**: Is the two-party percentage of the state’s electorate that voted for Hillary Clinton in 2016.

**Upcoming Governor Election**: An indicator for whether the incumbent governor is up for reelection in 2020 (1) or is not up for reelection (0).

**Total Interest Groups**: Is the total number of registered interest groups within a state. This variable is logged to make it more normally distributed. This data is from 2007. This data is from *The Correlates of State Policy Project*.

**Health Industry Interest Group**: Is the percentage of healthcare interest groups (i.e., health professionals associations, hospitals, and health insurers) in the state. This data is from 2007. This data is from *The Correlates of State Policy Project*.

**Hotel/Small Business Industry Interest Groups**: Is the percentage of small business, retail, hotel, and restaurant interest groups in a state. This data is from 2007. This data is from *The Correlates of State Policy Project*.

**New Fatalities**: This variable is the total daily deaths in a state. This variable is lagged by one day. This data is from *The COVID Tracking Project*.

**New Cases**: This variable is the total new daily positively identified COVID-19 cases in a state. This variable is lagged by one day. This data comes from *The COVID Tracking Project*.

**Income Per Capita**: This is a state’s income per capita. Higher values indicate a state is wealthier per capita. This data comes from the *American Community Survey*.

**Urban**: This is the percentage of the state that is classified as urban. This data is from the U.S. Census Bureau.

**Population Density**: This is a state’s population density. Higher values indicate a state is more densely populated.

**Percentage of African American**: This is the percentage of a state’s population that is African American.
*Source*: https://www.governing.com/gov-data/census/state-minority-population-data-estimates.html

**Percentage of Hispanic**: This is the percentage of a state’s population that is Hispanic.
*Source*: https://www.governing.com/gov-data/census/state-minority-population-data-estimates.html

**South**: This is a dichotomous variable indicating whether a state is in the South (1) or not (0). The following states are classified as southern: Alabama, Arkansas, Georgia, Florida, Louisiana, Mississippi, North Carolina, South Carolina, Tennessee, Texas, and Virginia.

**Duration**: This variable is a count of the number of days since President Trump declared a national emergency. I square this variable because it provides the best model fit.

**Appendix B: Alternative Duration Dependence**

In the main text, I control for duration dependence and square it. It is necessary to control for duration dependence to ensure that my results are not biased and are consistent. To demonstrate that my results are robust to alternative specifications of the duration dependence variable, I estimate a model with a linear time trend variable (no transformation) and with a logged time trend variable.

The results are shown below in Table B. The results are consistent with those presented in the manuscript. I rely on the model with the squared duration dependence variable because it provides the best model fit as indicated by the BIC score.

*Table B. Alternative Duration Dependence*

|  | Linear | Squared | Logged |  |  |
| --- | --- | --- | --- | --- | --- |
|  | Coefficient | Coefficient | Coefficient |  |  |
| Variable | (S.E.) | (S.E.) | (S.E.) |  |  |
| **Female Health Administrator** | **1.324*** | **1.246*** | **1.495*** |  |  |
|  | **(0.478)** | **(0.508)** | **(0.496)** |  |  |
| **Female Governor** | **-0.429** | **-0.613** | **-0556** |  |  |
|  | **(0.620)** | **(0.680)** | **(0.654)** |  |  |
| Democratic Governor | 1.549* | 1.635* | 1.788* |  |  |
|  | (0.517) | (0.523) | (0.529) |  |  |
| Democratic Electorate | 0.090* | 0.065* | 0.099* |  |  |
|  | (0.032) | (0.033) | (0.033) |  |  |
| Upcoming Governor Election | 1.320* | 1.572* | 1.578* |  |  |
|  | (0.602) | (0.605) | (0.623) |  |  |
| Total Interest Groups | -1.194* | -1.287* | -1.181* |  |  |
|  | (0.573) | (0.594) | (0.575) |  |  |
| Health Industry Interest Group | 0.078 | 0.045 | 0.082 |  |  |
|  | (0.111) | (0.119) | (0.114) |  |  |
| Hotel/Small Business Industry Interest Groups | -0.215 | -0.431* | -0.355* |  |  |
|  | (0.169) | (0.206) | (0.194) |  |  |
| New Fatalities | 0.103* | 0.094* | 0.088* |  |  |
|  | (0.040) | (0.044) | (0.041) |  |  |
| New Cases | 0.001 | 0.000 | 0.000 |  |  |
|  | (0.001) | (0.001) | (0.001) |  |  |
| Income Per Capita | -0.061* | -0.065* | -0.069* |  |  |
|  | (0.027) | (0.029) | (0.029) |  |  |
| Urban | -0.047* | -0.055* | -0.059* |  |  |
|  | (0.026) | (0.028) | (0.028) |  |  |
| Population Density | 0.001 | 0.003* | 0.002 |  |  |
|  | (0.001) | (0.001) | (0.001) |  |  |
| Percentage African American | 0.036 | 0.010 | 0.027 |  |  |
|  | (0.033) | (0.033) | (0.033) |  |  |
| Percentage Hispanic | 0.047* | 0.065* | 0.061* |  |  |
|  | (0.027) | (0.031) | (0.029) |  |  |
| South | -0.371 | -0.843 | -0.438 |  |  |
|  | (0.741) | (0.757) | (0.744) |  |  |
| Duration Dependence | 0.144* | 0.941* | ~ |  |  |
|  | (0.033) | (0.195) |  |  |  |
| Duration Dependence-Squared | ~ | -0.022* | ~ |  |  |
|  |  | (0.006) |  |  |  |
| Duration Dependence (Logged) | ~ | ~ | 3.094* |  |  |
|  |  |  | (0.623) |  |  |
| Constant | 0.348 | -1.379 | -4.495 |  |  |
|  | (5.372) | (5.852) | (5.681) |  |  |
| BIC | 394 | 364 | 373 | |  |
| Observations | 899 | 899 | 899 | |  |
| *Notes*: Coefficients are estimated with a logistic regression estimator. A random effect is included on each state. Bold entries indicate my key independent variables. **p* ≤ 0.05 (all one-tailed tests) | | | | | |
